# Case-control study of glucocorticoid receptor and corticotrophin-releasing hormone receptor gene variants and risk of perinatal depression

**DOI:** 10.1186/s12884-015-0720-z

**Published:** 2015-10-30

**Authors:** Ene-Choo Tan, Tze-Ern Chua, Theresa M. Y. Lee, Hui-San Tan, Joe L. Y. Ting, Helen Y. Chen

**Affiliations:** KK Research Centre, KK Women’s and Children’s Hospital, 100 Bukit Timah Road, Singapore, 229899 Singapore; Paediatrics Academic Clinical Programme, SingHealth Duke-NUS Graduate Medical School, 8 College Road, Singapore, 169857 Singapore; Department of Psychological Medicine, KK Women’s and Children’s Hospital, 100 Bukit Timah Road, Singapore, 229899 Singapore

**Keywords:** Genetic association, Family history, Menstrual period regularity, Perinatal depression, Pregnancy

## Abstract

**Background:**

Depression during pregnancy or after childbirth is the most frequent perinatal illness affecting women of reproductive age. It could result in unfavourable outcomes for both women and their newborns. The incidence of perinatal depression is higher for those with family history of depression and other mental illness, suggesting the contribution of genetic factors. There is postulation that disruption or fluctuation of reproductive hormones could play a part in women who are sensitive to such changes.

**Methods:**

This is a case-control study comparing the frequencies of candidate gene variants in patients with perinatal depression with controls. Patients of Chinese descent (*N* = 725) were recruited from the outpatient clinics of the hospital between 2010 and 2013. Controls were patients who came for postnatal consultations at the obstetrics clinics and scored ≤ 7 on the Edinburgh Postnatal Depression Scale (EPDS) at the postnatal screening programme of the hospital. Cases with confirmed diagnosis of clinical (major) depression related to pregnancy/postpartum were recruited from the hospital’s outpatient clinic. Genomic DNA was extracted from saliva samples and genotyped for the polymorphisms of interest. Differences between groups were assessed by chi-square analysis.

**Results:**

*CRHR1* rs242939 and rs1876828 were not polymorphic in the study population. There was no statistically significant association of perinatal depression for *CRHR1* rs242941 and *GR* rs41423247 (*Bcl*I). When all subjects were grouped based on family history of mental illness, there was a statistically significant association of *CRHR1* rs242941 with family history regardless of depression status (*P* = 0.043). There was also a statistically significant difference for *GR* rs41423247 and regularity of menstrual periods (*P* < 0.000). Although not statistically significant, women with perinatal depression showed a trend towards higher frequency of self-reported menstrual irregularity.

**Conclusions:**

No evidence was found for the association of any of the genetic markers with perinatal depression in this study cohort. Instead, the possible genetic links were found in women with positive family history of mental illness and menstrual irregularity, suggesting these could be identifying risk markers for women.

## Background

Depression is a complex heterogeneous disorder that results from gene-environment interaction [1, 2]. Evidence for genetic contribution comes from twin and family studies, which suggests a heritability of 30 – 70 % and a 3-fold increased risk for first-degree relatives [3].

Depression affects all populations worldwide and is a major cause of disability and loss of productivity. The prevalence in women is higher than that of men. Women of childbearing age have the highest risk of developing depression, with close to 20 % suffering from postnatal depression or having depressive symptoms during pregnancy [4, 5]. The prevalence of antenatal depression in Singapore is between 12 % and 20 % and for postnatal depression it is 6.8 % [6, 7]. This rate is close to the range reported for western populations [8–10].

There is evidence that in some women, childbirth may be a specific trigger for depressive illness. Previous studies have proposed that the development of depression in some women could be triggered by striking changes in hormonal levels during pregnancy and following childbirth. These hormones include oestrogen, progesterone, corticotrophin-releasing hormone, cortisol and glucocorticoids [11–15]. In a genome-wide linkage study, the strongest signals that may be specific to postpartum mood symptoms were on 1q21.3-q32.1 and 9p24.3-p22.3, with modest association for Hemicentin gene SNPs on chromosome 1 [16]. The gene might have oestrogen receptor binding sites and was highly expressed in the hippocampus region. Drop in oestrogen levels after a hormone-stimulated pregnancy has been reported to alter hippocampal cell proliferation in rats [17]. Among transcripts which showed different expression patterns between women with postpartum depression and euthymic women, there was enrichment of those implicated in the oestrogen signalling pathway [18].

Disruption in ovarian function manifested as a change in menstrual cycle length has been linked to higher cardio-metabolic risk, higher scores for Center for Epidemiological Studies Depression Scale, and higher likelihood to have had a diagnosis of depression or used anti-depressant medication [19]. A link between psychiatric disorders and regularity of menstrual cycle has also been found in Caucasian women [20].

Past history of depression and family history of mental illness remain the two strongest predictors of perinatal depression [21]. Given the role of the hypothalamic-pituitary-adrenocortical (HPA) axis in the aetiology of depression and the changing levels of corticotrophin-releasing hormone (*CRHR1*) during pregnancy, it is likely that the predisposition to perinatal depression might be related to genetic polymorphisms in the hormone receptors. Indeed, several studies have shown that polymorphisms at the glucocorticoid receptor (*GR*), such as *Bcl*I and ER22/23EK were associated with dysfunction of the HPA axis and altered glucocorticoid sensitivity leading to depression [22–25]. Single nucleotide polymorphisms and haplotypes of *CRHR1* were also found to be significantly over-represented in patients with major depression compared to controls [26–28].

Several studies have looked into specific single nucleotide polymorphisms (SNPs) of the glucocorticoid and corticotrophin-releasing hormone receptor genes in relation to the risk of developing depression during pregnancy and the postnatal period. The results range from none [29, 30] to very strong positive association with risk ratios of 2.9 – 5.48 [31].

In this study, we investigated the five SNPs in the study which showed high risk ratios [31] in a cohort of hospital patients that included controls who had been screened for postnatal depression and cases who met diagnostic criteria for perinatal depression. Besides providing replication for the positive association, our aim was also to obtain the genotypic frequencies of the five SNPs, namely *Bcl*I (rs41423247) and ER22/23EK (rs6189–6190) of *GR;* and rs242941, rs242939 and rs1876828 of *CRHR1* in our Singaporean Chinese population.

## Methods

### Study participants

The study design was reviewed and approved by the SingHealth Centralised Institutional Review Board which oversees all research studies in the hospital. The investigation was carried out in accordance with the latest version of the Declaration of Helsinki. Written informed consent of the participants was obtained after the procedures had been fully explained to them. The study sample has been described in a previous report [32]. In short, cases were recruited from outpatient psychiatric clinics and were all diagnosed by board-certified psychiatrists according to the criteria in Diagnostic Statistical Manual of Mental Disorders IV edition. Inclusion criteria for cases were being of Chinese ancestry and having a confirmed diagnosis of clinical (major) depression related to pregnancy/postpartum. However, those with comorbid predominant anxiety, psychotic disorders, or substance abuse were excluded from the analysis. Controls were recruited from patients who attended postnatal obstetrics clinics and were screened for absence of symptoms of postnatal depression using the Edinburgh Postnatal Depression Scale (EPDS). Only those with EPDS score ≤ 7 and self-reported as being Chinese were included. From a cohort of 725 subjects recruited between November 2010 and March 2013, 147 cases and 549 controls of self-reported Chinese ancestry were included in this study.

Data on demographics, personal and family history of mental illness, menstrual cycle regularity and mood changes were captured through in-person interviews based on a standard set of questions and recorded on a data collection form at the time of recruitment.

### Molecular analysis

DNA was extracted from saliva samples using either the Oragene DNA Kit (DNA Genotek Inc., Kanata, Canada) or the Norgen Saliva DNA collection kit (Norgen Biotek Corp., Thorold, Canada). DNA was quantitated using Nanodrop Spectrophotometer (Thermo Scientific, Wilmington, USA).

Genotyping of the three *CRHR1* SNPs were done using Taqman assays: rs242941 - Assay ID: AHLJYBU; rs242939 - Assay ID: C__2544833_10, rs1876828 - Assay ID: 11935972_10. (Applied Biosystems, Foster City, CA, USA). Amplification was performed in a volume of 12 μl containing 25 ng genomic DNA, Taqman Universal Polymerase Chain Reaction Master Mix, 60 nM of each probe, and 270 nM of each primer. Cycling and hybridization conditions were set according to manufacturer’s instructions. The 50 cycles of denaturation and annealing/extension and post-polymerase chain reaction quantification of fluorescent intensity were performed using the Applied Biosystems StepOnePlus Real-Time PCR System. Alleles were called using the sequence detection software.

Genotyping of the *GR Bcl*I (rs41423247) was done by polymerase chain reaction followed by restriction with *Bcl*I and electrophoresis on 3 % agarose gel. Genotypes were scored manually. No genotyping was performed for *GR* ER22/23EK (rs6189–6190) because multiple studies show that it is not polymorphic in the Chinese population [33–35].

### Statistical analysis

For genotype distribution, deviation from Hardy-Weinberg equilibrium (HWE) was evaluated using a chi-square (*χ*^2^) test. Difference in distribution of categorical variables between groups was also assessed by *χ*^2^ test. All analysis was performed using Statistical Package for the Social Sciences (SPSS, IBM Corp., Armonk, NY, USA), version 19.0, with statistical significance set at 0.05.

## Results

For two of the three polymorphisms investigated for the *CRHR1* gene, all samples that were genotyped (>100 individuals) for SNPs rs242939 and rs1876828 were homozygous for the major allele (AA and CC respectively). Genotyping was not performed for the remaining samples. Genotyping error was ruled out by using samples of Indian and Caucasian ancestry, with the successful detection of the minor alleles for both SNPs.

Results for the remaining two SNPs for cases and controls are presented in Table 1. Genotyping was successful for 99.7 % of the samples for *CRHR1* rs242941 and 98.9 % for *GR* rs41423247. Genotype distributions for both SNPs were in Hardy-Weinberg equilibrium. There was no difference in the distribution between the case and control groups.

**Table 1:** Genotype distribution for controls and cases for the two polymorphic variants

Gene	Genotypes	Controls (%)	Cases (%)	*χ* ^2^	*P*-value
*CRHR1*	rs242941 GG	472 (86.0)	127 (86.4)		
	rs242941 GT	71 (12.9)	19 (12.9)	0.199	0.905
	rs242941 TT	6 (1.1)	1 (0.7)		
*GR*	rs41423247 GG	326 (60.0)	88 (59.9)		
	rs41423247 GC	194 (35.7)	50 (34.0)	0.985	0.611
	rs41423247 CC	23 (4.2)	9 (6.1)		

CRHR1: *N* = 696 GR *N* = 690

When the diagnosis of depression was not considered, there was a statistically significant association between *CRHR1* rs242941 and family history of mental illness (Table 2); and between *GR* rs41423247 and regularity of menstrual periods (Table 3). Among the cases with perinatal depression, there was a higher proportion (22.5 %) who self-reported that their menstrual cycles were not regular, compared to 17.3 % among controls; although this was not statistically significant (*χ*^2^ = 3.123, *P* = 0.210).

**Table 2:** Genotype distribution for the two polymorphisms and family history (FH) of mental illness

Gene	Genotypes	No FH (%)	Positive FH (%)	*χ* ^2^	*P*-value
*CRHR1*	rs242941 GG	536 (87.0)	63 (78.8)		
	rs242941 GT	73 (11.9)	17 (21.3)	6.308	0.043
	rs242941 TT	7 (1.1)	0 (0.0)		
*GR*	rs41423247 GG	368 (60.3)	46 (57.5)		
	rs41423247 GC	217 (35.6)	27 (33.8)	3.461	0.177
	rs41423247 CC	25 (4.1)	7 (8.8)

**Table 3:** Genotype distribution for the two polymorphisms and regularity of menstrual cycles

Gene	Genotypes	Regular (%)	Not regular (%)	*χ* ^2^	*P*-value
*CRHR1*	rs242941 GG	481 (85.0)	117 (90.7)		
	rs242941 GT	79 (14.0)	11 (8.5)	2.876	0.237
	rs242941 TT	6 (1.1)	1 (0.8)		
*GR*	rs41423247 GG	354 (63.2)	59 (45.7)		
	rs41423247 GC	188 (33.6)	56 (43.4)	21.381	0.000
	rs41423247 CC	18 (3.2)	14 (10.9)		

## Discussion

During pregnancy, the development of a transient organ of foetal origin, the placenta, causes major alterations in the hypothalamic-pituitary-adrenal (HPA) axis. It has been suggested that HPA axis dysregulation during this period of dramatic change in hormonal levels could cause the development of postpartum depression in vulnerable women [36]. Life stress was also found to be an important risk factor for depressive symptoms during pregnancy in a systematic review of 57 studies [37]. Genes related to HPA axis and stress reactivity are thus good candidate genes for perinatal depression.

A recent study by Engineer et al. found that the *Bcl*I single nucleotide polymorphism of the *GR* gene and the rs242939 SNP of the *CRHR1* gene were associated with genetic risk to antenatal and postnatal depression [31]. We found no evidence of an association between the *GR Bcl*I SNP and perinatal depression while this *CRHR1* SNP is not polymorphic in our population. Engineer et al. also reported that the G-G-T haplotype of the *CRHR1* was significantly over-represented in patients with high EPDS depression scores. We were not able to do a similar haplotype analysis because two of the three markers (*CRHR1* rs242939 and rs1876828) were not polymorphic in our population.

Compared to the study, our cohort has almost five times the number of study subjects (*N* = 696 versus *N* = 140), with higher power to detect a statistically significant difference. There are also two major differences between our study and that of Engineer et al. [31]. One is the ethnic origin of the population studied. Our cohort comprised Chinese women while theirs was a Western population. However, the prevalence of antenatal and postnatal depression in Singapore is similar to the range for Western population; with no evidence of decreased susceptibility to perinatal depression in our population. Another difference between the two studies is the method of classification of the study subjects. Although both were prospective studies involving a hospital population, ours is a case-control design. Controls were screened with EPDS while cases were patients who had been evaluated by psychiatrists as meeting DSM IV criteria for depression; whereas Engineer’s cohort were divided into two groups with low versus high postnatal depression risk. In their study, women who scored below 10 on the EPDS were placed in the “Low PND Risk” group and those with EPDS of 10 and above comprised the “High PND Risk” group. There was no clinical assessment on the symptoms of the subjects; while we use EPDS ≤ 7 for controls and clinically diagnosed depression for cases. As EPDS is a screening rather than a diagnostic tool, using it to classify the subjects might not reflect the actual risk status of the patients.

Another group from the UK also attempted to replicate the above findings using an existing cohort. As the *GR* SNPs *Bcl*I and ER22/23EK (rs41423247 and rs6190 respectively) used in the original study were not available in the dataset from the genotyping platform used (Illumina human 660 W-quad genome-wide SNP genotyping platform), two other SNPs which are in strong linkage disequilibrium with the respective SNPs – rs853180 (r^2^ = 0.965 with rs41423247) and rs10482704 (r^2^ = 0.793 with rs6190) – were used for analysis. Logistic regression analysis with EPDS scores as a continuous variable found no association of any of the five SNPs with EPDS scores at 18 weeks gestation, and weak association of three of the SNPs (*GR* rs853180 and rs10482704; *CRHR1* rs242939) with EPDS scores at 8 weeks postnatal. Analysing it as a binary variable (which was used in ours and Engineer’s studies) showed similar results [30]. Another group from Germany also found no association with the *Bcl*I SNP or two other *GR* SNPS (rs6195 and rs10482605), or two *CRHR1* SNPs (rs110402 and rs7209436) or any haplotype tested [29].

Although our study on a Chinese population found no evidence of association of the specific *GR* and *CRHR1* SNPs and risk of pregnancy-related depression in our population, there was a statistically significant association with positive family history for *CRHR1* rs242941. This SNP has been found to constitute part of a haplotype predisposing to depression and anti-depressant responsiveness [28, 38, 39].

For *GR* rs41423247 (*Bcl*I), although we found no evidence of association with a diagnosis of perinatal depression, there was a highly significant association with the regularity of menstrual cycles. Genotypes containing the minor allele (C) were more common among patients who self-reported that their menstrual cycles were not regular. The variant has been found to be associated with increased glucocorticoid sensitivity [40, 41]. This genotype with the C allele and/or C-containing genotypes have also been found to be associated with depression or higher depression scores [22, 42–44].

A main limitation of our study is the lack of data regarding the exact timing of onset of perinatal depression, whether cases had their onset during pregnancy or in the postnatal period. However, there is good evidence for genetic links in depression with onset at both times. Additionally, there is good evidence of the close link between antenatal and postnatal onset depression, with 23 – 45 % postnatally depressed women having antenatal depression [45, 46]. Other limitations were that the patterns of change in menstrual cycle length and mood symptoms were self-reported and retrospective with women reporting changes in a typical cycle over the past 12 months.

## Conclusions

There is no association between the specific SNPs and the diagnosis of perinatal depression in this cohort of Singaporean Chinese patients. However, based on the our findings on the associations with family history of mental illness and menstrual period regularity, the GR and CHR1 pathways may still be involved in the predisposition to mental illness, and menstrual period regularity may be a predisposing factor in susceptible women.

There is a need for more studies in different populations to find the genetic factors associated with depression during pregnancy and following delivery. Identification of biomarkers facilitates screening of at-risk individuals for early intervention, choice of treatment, prediction of treatment response, and prognosis of outcome over a wide spectrum of symptoms associated with affective states, thereby optimizing clinical practice procedures.

## References

[1] Klengel T, Binder EB (2013). Gene-environment interactions in major depressive disorder. Can J Psychiatry.

[2] Mandelli L, Serretti A (2013). Gene environment interaction studies in depression and suicidal behavior: An update. Neurosci Biobehav Rev.

[3] Lohoff FW (2010). Overview of the genetics of major depressive disorder. Curr Psychiatry Rep.

[4] Gavin NI, Gaynes BN, Lohr KN, Meltzer-Brody S, Gartlehner G, Swinson T (2005). Perinatal depression: a systematic review of prevalence and incidence. Obstet Gynecol.

[5] Mann R, Gilbody S, Adamson J (2010). Prevalence and incidence of postnatal depression: what can systematic reviews tell us?. Arch Womens Ment Health.

[6] Chee CY, Lee DT, Chong YS, Tan LK, Ng TP, Fones CS (2005). Confinement and other psychosocial factors in perinatal depression: a transcultural study in Singapore. J Affect Disord.

[7] Chen H, Chan YH, Tan KH, Lee T (2004). Depressive symptomatology in pregnancy - a Singaporean perspective. Soc Psychiatry Psychiatr Epidemiol.

[8] Evans J, Heron J, Francomb H, Oke S, Golding J (2001). Cohort study of depressed mood during pregnancy and after childbirth. BMJ.

[9] Gorman LL, O’Hara MW, Figueiredo B, Hayes S, Jacquemain F, Kammerer MH, Klier CM, Rosi S, Seneviratne G, Sutter-Dallay AL (2004). Adaptation of the structured clinical interview for DSM-IV disorders for assessing depression in women during pregnancy and post-partum across countries and cultures. Br J Psychiatry Suppl.

[10] Setse R, Grogan R, Pham L, Cooper LA, Strobino D, Powe NR, Nicholson W (2009). Longitudinal study of depressive symptoms and health-related quality of life during pregnancy and after delivery: the Health Status in Pregnancy (HIP) study. Matern Child Health J.

[11] Kammerer M, Taylor A, Glover V (2006). The HPA axis and perinatal depression: a hypothesis. Arch Womens Ment Health.

[12] O’Keane V, Lightman S, Patrick K, Marsh M, Papadopoulos AS, Pawlby S, Seneviratne G, Taylor A, Moore R (2011). Changes in the maternal hypothalamic-pituitary-adrenal axis during the early puerperium may be related to the postpartum ‘blues’. J Neuroendocrinol.

[13] Glynn LM, Sandman CA (2014). Evaluation of the association between placental corticotrophin-releasing hormone and postpartum depressive symptoms. Psychosom Med.

[14] Yim IS, Glynn LM, Dunkel-Schetter C, Hobel CJ, Chicz-DeMet A, Sandman CA (2009). Risk of postpartum depressive symptoms with elevated corticotropin-releasing hormone in human pregnancy. Arch Gen Psychiatry.

[15] Bloch M, Rubinow DR, Schmidt PJ, Lotsikas A, Chrousos GP, Cizza G (2005). Cortisol response to ovine corticotropin-releasing hormone in a model of pregnancy and parturition in euthymic women with and without a history of postpartum depression. J Clin Endocrinol Metab.

[16] Mahon PB, Payne JL, MacKinnon DF, Mondimore FM, Goes FS, Schweizer B, Jancic D, Coryell WH, Holmans PA, Shi J (2009). Genome-wide linkage and follow-up association study of postpartum mood symptoms. Am J Psychiatry.

[17] Green AD, Galea LA (2008). Adult hippocampal cell proliferation is suppressed with estrogen withdrawal after a hormone-simulated pregnancy. Horm Behav.

[18] Mehta D, Newport DJ, Frishman G, Kraus L, Rex-Haffner M, Ritchie JC, Lori A, Knight BT, Stagnaro E, Ruepp A (2014). Early predictive biomarkers for postpartum depression point to a role for estrogen receptor signaling. Psychol Med.

[19] Bleil ME, Bromberger JT, Latham MD, Adler NE, Pasch LA, Gregorich SE, Rosen MP, Cedars MI (2013). Disruptions in ovarian function are related to depression and cardiometabolic risk during premenopause. Menopause.

[20] Barron ML, Flick LH, Cook CA, Homan SM, Campbell C (2008). Associations between psychiatric disorders and menstrual cycle characteristics. Arch Psychiatr Nurs.

[21] Figueiredo FP, Parada AP, Araujo LF, Silva WA, Del Ben CM (2014). The Influence of genetic factors on peripartum depression: A systematic review. J Affect Disord.

[22] Spijker AT, van Rossum EF (2009). Glucocorticoid receptor polymorphisms in major depression. Focus on glucocorticoid sensitivity and neurocognitive functioning. Ann N Y Acad Sci.

[23] Derijk RH, de Kloet ER (2008). Corticosteroid receptor polymorphisms: determinants of vulnerability and resilience. Eur J Pharmacol.

[24] Szczepankiewicz A, Leszczynska-Rodziewicz A, Pawlak J, Rajewska-Rager A, Dmitrzak-Weglarz M, Wilkosc M, Skibinska M, Hauser J (2011). Glucocorticoid receptor polymorphism is associated with major depression and predominance of depression in the course of bipolar disorder. J Affect Disord.

[25] Claes S (2009). Glucocorticoid receptor polymorphisms in major depression. Ann N Y Acad Sci.

[26] Claes S, Villafuerte S, Forsgren T, Sluijs S, Del-Favero J, Adolfsson R, Van Broeckhoven C (2003). The corticotropin-releasing hormone binding protein is associated with major depression in a population from Northern Sweden. Biol Psychiatry.

[27] Ishitobi Y, Nakayama S, Yamaguchi K, Kanehisa M, Higuma H, Maruyama Y, Ninomiya T, Okamoto S, Tanaka Y, Tsuru J (2012). Association of CRHR1 and CRHR2 with major depressive disorder and panic disorder in a Japanese population. Am J Med Genet B Neuropsychiatr Genet.

[28] Liu Z, Zhu F, Wang G, Xiao Z, Wang H, Tang J, Wang X, Qiu D, Liu W, Cao Z, Li W (2006). Association of corticotropin-releasing hormone receptor1 gene SNP and haplotype with major depression. Neurosci Lett.

[29] Schneider M, Engel A, Fasching PA, Haberle L, Binder EB, Voigt F, Grimm J, Faschingbauer F, Eichler A, Dammer U (2014). Genetic variants in the genes of the stress hormone signalling pathway and depressive symptoms during and after pregnancy. Biomed Res Int.

[30] Stergiakouli E, Sterne JA, Smith GD (2014). Failure to replicate the association of glucocorticoid and type 1 corticotropin-releasing hormone receptors gene variants with risk of depression during pregnancy and post-partum reported by. J Psychiatr Res.

[31] Engineer N, Darwin L, Nishigandh D, Ngianga-Bakwin K, Smith SC, Grammatopoulos DK (2013). Association of glucocorticoid and type 1 corticotropin-releasing hormone receptors gene variants and risk for depression during pregnancy and post-partum. J Psychiatr Res.

[32] Tan EC, Tan HS, Chua TE, Lee T, Ng J, Ch’ng YC, Choo CH, Chen HY (2014). Association of premenstrual/menstrual symptoms with perinatal depression and a polymorphic repeat in the polyglutamine tract of the retinoic acid induced 1 gene. J Affect Disord.

[33] Duan ZX, Gu W, Du DY, Hu P, Jiang DP, Zhu PF, Wang ZG, Jiang JX (2009). Distributions of glucocorticoid receptor gene polymorphisms in a Chinese Han population and associations with outcome after major trauma. Injury.

[34] Wang LL, Xie YC, Hou SF, Feng K, Yin J, Xu XH, Li ZB, Wang YP, Xiong T, Liu JJ, Shen DG (2009). [Association of glucocorticoid receptor gene polymorphism with myasthenia gravis]. Zhonghua Yi Xue Za Zhi.

[35] Xuan M, Li H, Fu R, Yang Y, Zhang D, Zhang X, Yang R (2014). Lack of association between NR3C1 polymorphism and glucocorticoid resistance in Chinese patients with immune thrombocytopenia. Platelets.

[36] Glynn LM, Davis EP, Sandman CA (2013). New insights into the role of perinatal HPA-axis dysregulation in postpartum depression. Neuropeptides.

[37] Lancaster CA, Gold KJ, Flynn HA, Yoo H, Marcus SM, Davis MM (2010). Risk factors for depressive symptoms during pregnancy: a systematic review. Am J Obstet Gynecol.

[38] Licinio J, O’Kirwan F, Irizarry K, Merriman B, Thakur S, Jepson R, Lake S, Tantisira KG, Weiss ST, Wong ML (2004). Association of a corticotropin-releasing hormone receptor 1 haplotype and antidepressant treatment response in Mexican-Americans. Mol Psychiatry.

[39] Liu Z, Zhu F, Wang G, Xiao Z, Tang J, Liu W, Wang H, Liu H, Wang X, Wu Y (2007). Association study of corticotropin-releasing hormone receptor1 gene polymorphisms and antidepressant response in major depressive disorders. Neurosci Lett.

[40] Manenschijn L, Van Den Akker EL, Lamberts SW, Van Rossum EF (2009). Clinical features associated with glucocorticoid receptor polymorphisms. Overview Ann N Y Acad Sci.

[41] Wust S, Van Rossum EF, Federenko IS, Koper JW, Kumsta R, Hellhammer DH (2004). Common polymorphisms in the glucocorticoid receptor gene are associated with adrenocortical responses to psychosocial stress. J Clin Endocrinol Metab.

[42] van Rossum EF, Binder EB, Majer M, Koper JW, Ising M, Modell S, Salyakina D, Lamberts SW, Holsboer F (2006). Polymorphisms of the glucocorticoid receptor gene and major depression. Biol Psychiatry.

[43] Galecka E, Szemraj J, Bienkiewicz M, Majsterek I, Przybylowska-Sygut K, Galecki P, Lewinski A (2013). Single nucleotide polymorphisms of NR3C1 gene and recurrent depressive disorder in population of Poland. Mol Biol Rep.

[44] Reuter M, Markett S, Melchers M, Montag C (2012). Interaction of the cholinergic system and the hypothalamic-pituitary-adrenal axis as a risk factor for depression: evidence from a genetic association study. Neuroreport.

[45] Green JM (1998). Postnatal depression or perinatal dysphoria? Findings from a longitudinal community-based study using the Edinburgh Postnatal Depression Scale. J Reprod Infant Psychol.

[46] Watson JP, Elliott SA, Rugg AJ, Brough DI (1984). Psychiatric disorder in pregnancy and the first postnatal year. Br J Psychiatry.

